# Corticotropin-releasing factor infusion in the bed nucleus of the stria terminalis of lactating mice alters maternal care and induces behavioural phenotypes in offspring

**DOI:** 10.1038/s41598-020-77118-7

**Published:** 2020-11-17

**Authors:** Kerstin Camile Creutzberg, Érika Kestering-Ferreira, Thiago Wendt Viola, Luis Eduardo Wearick-Silva, Rodrigo Orso, Bernardo Aguzzoli Heberle, Lucas Albrechet-Souza, Rosa Maria Martins de Almeida, Rodrigo Grassi-Oliveira

**Affiliations:** 1 Developmental Cognitive Neuroscience Lab, Brain Institute of Rio Grande Do Sul (InsCer), Pontifical Catholic University of Rio Grande Do Sul (PUCRS), Jardim Botânico, Porto Alegre, RS Brazil; 2 Department of Physiology, Louisiana State University Health Sciences Center, New Orleans, LA USA; 3 Psychology Institute, Federal University of Rio Grande Do Sul, Porto Alegre, RS Brazil

**Keywords:** Developmental biology, Disease model

## Abstract

The peripartum period is accompanied by numerous physiological and behavioural adaptations organised by the maternal brain. These changes are essential for adequate expression of maternal behaviour, thereby ensuring proper development of the offspring. The corticotropin-releasing factor (CRF) plays a key role in a variety of behaviours accompanying stress, anxiety, and depression. There is also evidence that CRF contributes to maladaptations during the peripartum period. We investigated the effects of CRF in the bed nucleus of the stria terminalis (BNST) of lactating mice during maternal care and analysed locomotor activity and anxiety-like behaviour in the offspring. The BNST has been implicated in anxiety behaviour and regulation of the stress response. The effects of intra-BNST CRF administration were compared with those induced by the limited bedding (LB) procedure, a model that produces altered maternal behaviour. BALB/cJ dams were exposed to five infusions of CRF or saline into the BNST in the first weeks after birth while the LB dams were exposed to limited nesting material from postnatal days (P) 2–9. Maternal behaviour was recorded in intercalated days, from P1-9. Offspring anxiety-like behaviour was assessed during adulthood using the open-field, elevated plus-maze, and light/dark tests. Both intra-BNST CRF and LB exposure produced altered maternal care, represented by decreased arched-back nursing and increased frequency of exits from the nest. These changes in maternal care resulted in robust sex-based differences in the offspring’s behavioural responses during adulthood. Females raised by CRF-infused dams exhibited increased anxiety-like behaviour, whereas males presented a significant decrease in anxiety. On the other hand, both males and females raised by dams exposed to LB showed higher locomotor activity. Our study demonstrates that maternal care is impaired by intra-BNST CRF administrations, and these maladaptations are similar to exposure to adverse early environments. These procedures, however, produce distinct phenotypes in mice during young adulthood and suggest sex-based differences in the susceptibility to poor maternal care.

## Introduction

Childhood is considered to be a critical period for the development of an individual^1^. Due to the many changes that the central nervous system undergoes during early life, it can be influenced both positively or negatively by environmental factors^2^. Exposure to adverse events early in life, such as poor maternal care, neglect, and poverty, can lead to long-lasting neurobiological and behavioural consequences, such as altered glucocorticoid signalling, functional and structural changes in the brain, and altered nociceptive behaviour^3–5^. Moreover, early life stress exposure increases the risk for the development of several psychiatric disorders later in life^6,7^.

Anxiety disorders, such as panic, distinct phobias, and generalised anxiety, are frequent debilitating problems^8,9^. In 2017, the World Health Organisation reported that 264 million people (3.6% of the global population) suffered from anxiety disorders worldwide^10^. These disorders have higher rates of comorbidity than other mental and physical diseases; 38.8% of patients with schizophrenia and 85% of those with depression disorders present comorbid anxiety^11,12^. For this reason, these disorders have higher social and economic burden^13–15^. Childhood trauma and genetic predisposition are considered major risk factors for developing a psychiatric disorder later in life^9,13,15^, and epidemiological studies have shown that people exposed to emotional abuse and neglect during childhood are at a greater risk of developing anxiety and post-traumatic stress disorders^16,17^.

Preclinical models are important tools for the investigation of neurobiological mechanisms underlying the relationship between early life stress and anxiety-related behaviours later in life. Accumulating evidence indicates that rodents exposed to a model of impoverished environment during early life showed fragmented maternal care, increased plasma corticosterone levels, and altered offspring risk-taking and anxiety-like behaviour^18–20^. These consequences seem to be attributed in part to low levels of maternal care^21^, given that the quality of the relationship between dam and pup during the first weeks of life has been used as a possible predictor of offspring differences later in life^22^.

A previous study demonstrated that the infusion of corticotropin-releasing factor (CRF) in the bed nucleus of the stria terminalis (BNST) alters maternal care^23^. This alteration is driven mainly by a decrease in arched-back nursing (ABN), which is considered an essential maternal care behaviour during the first weeks of life for adequate pup neurodevelopment^24–26^. The primary role of CRF is the activation of the hypothalamic–pituitary–adrenal (HPA) axis^27,28^, but it is also involved in neural, endocrine, and immunologic processes. CRF binds to two distinct receptors, namely, CRF receptor types 1 (CRFR1) and 2, with higher affinity for CRFR1. Moreover, CRF is highly present in hypothalamic areas, and axons of CRF-containing neurons project to extrahypothalamic areas, including the amygdala, ventral tegmental area, and BNST^29–31^. The BNST is a complex brain region that has a critical role in stress responses and stress-related disorders, including anxiety^32,33^. BNST expresses both CRF receptors and has been implicated in maternal care^32,34^.

**Figure 1:**
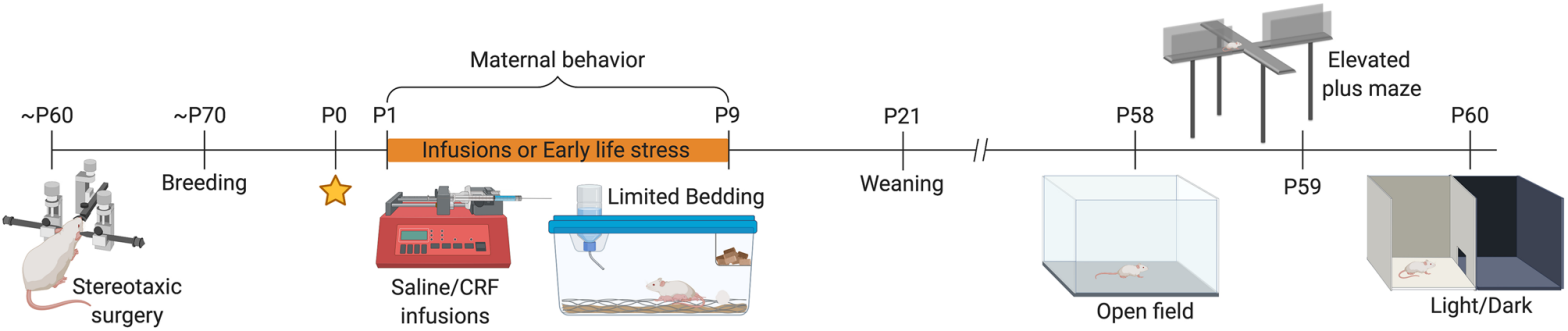
Experimental design (created with BioRender.com).

**Figure 2:**
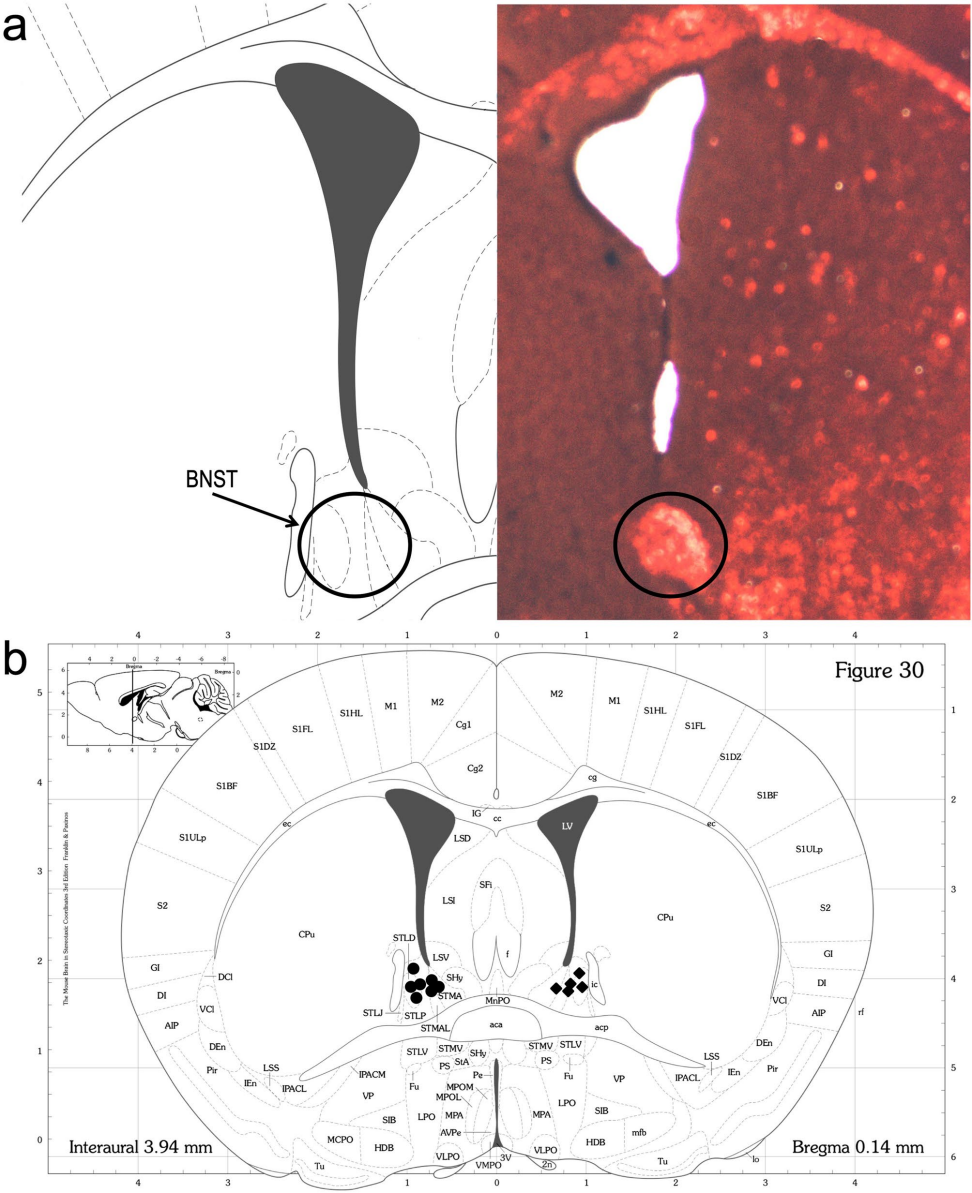
Cannula placement. (**a**) Representative image after staining (haematoxylin and eosin). (**b**) Placement of the cannula in a coronal section of the mouse brain^66^; circles for saline-infused (SAL) dams and diamonds for corticotropin-releasing factor-infused (CRF) dams.

To the best of our knowledge, no study has investigated the impact of CRF infusion into the BNST of dams on maternal care and subsequent behavioural changes of the offspring during adulthood. We compared the effects of this pharmacological manipulation with those produced by an environmental stressor – limited bedding (LB) – during the same period of life, which is known to induce altered maternal care. In this study, CRF infusions into the BNST impaired maternal care without the addition of environmental stress to induce alterations of murine dam-pup interactions (Figs. 1, 2).

## Results

### Maternal behaviour

**Figure 3:**
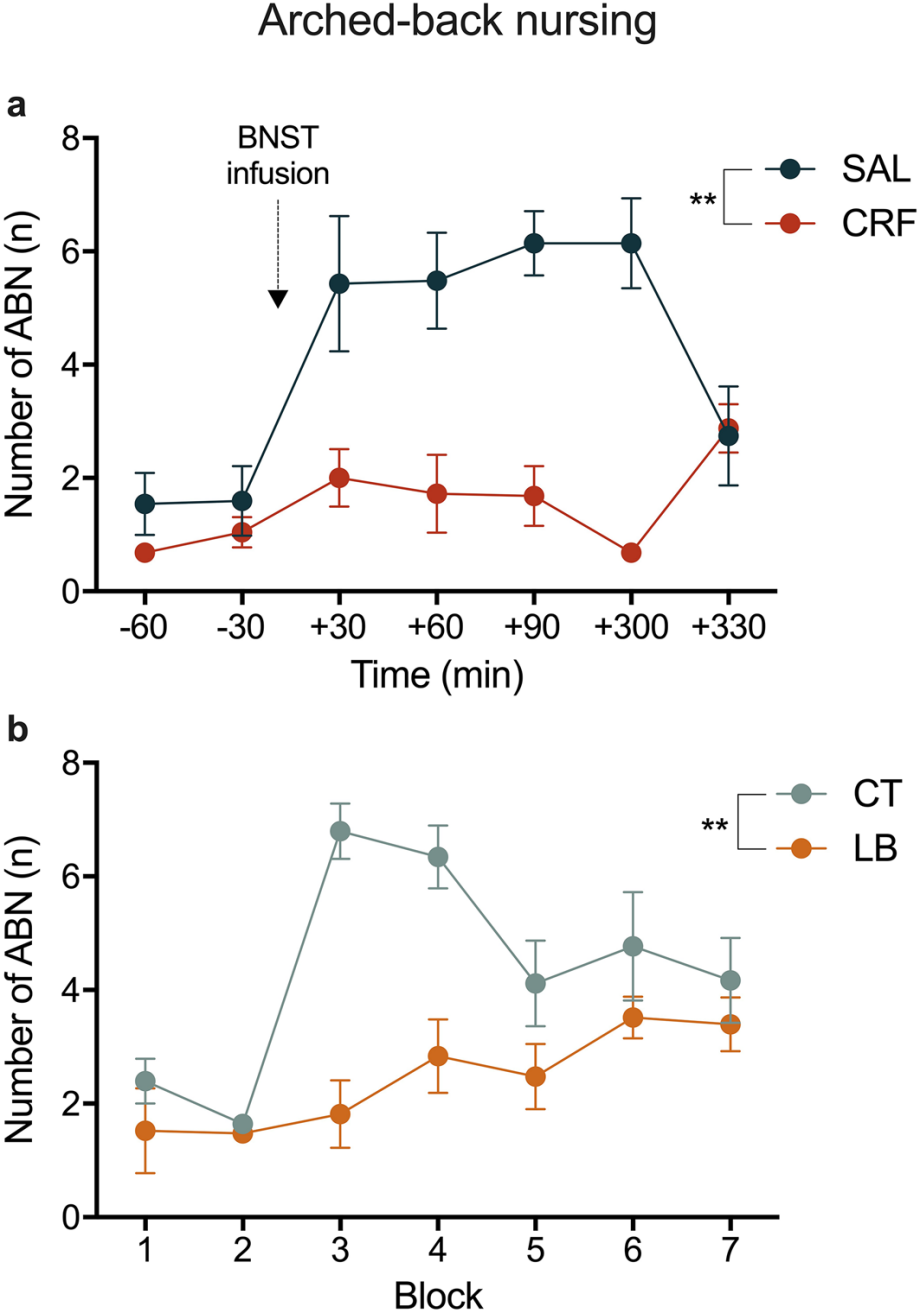
Maternal behaviour: Arched-back nursing. (**a**) Frequency of ABN in SAL and CRF dams per block. (**b**) Frequency of ABN in CT and LB dams per block. Data are presented as means SEM of all days from each block. **p < 0.01, repeated-measures ANOVA, treatment effect. *n* = 5–7 dams per group. SAL, saline-infused dams; CRF, corticotropin-releasing factor-infused dams; CT, control dams; LB, stressed dams; ABN, arched-back nursing.

**Figure 4:**
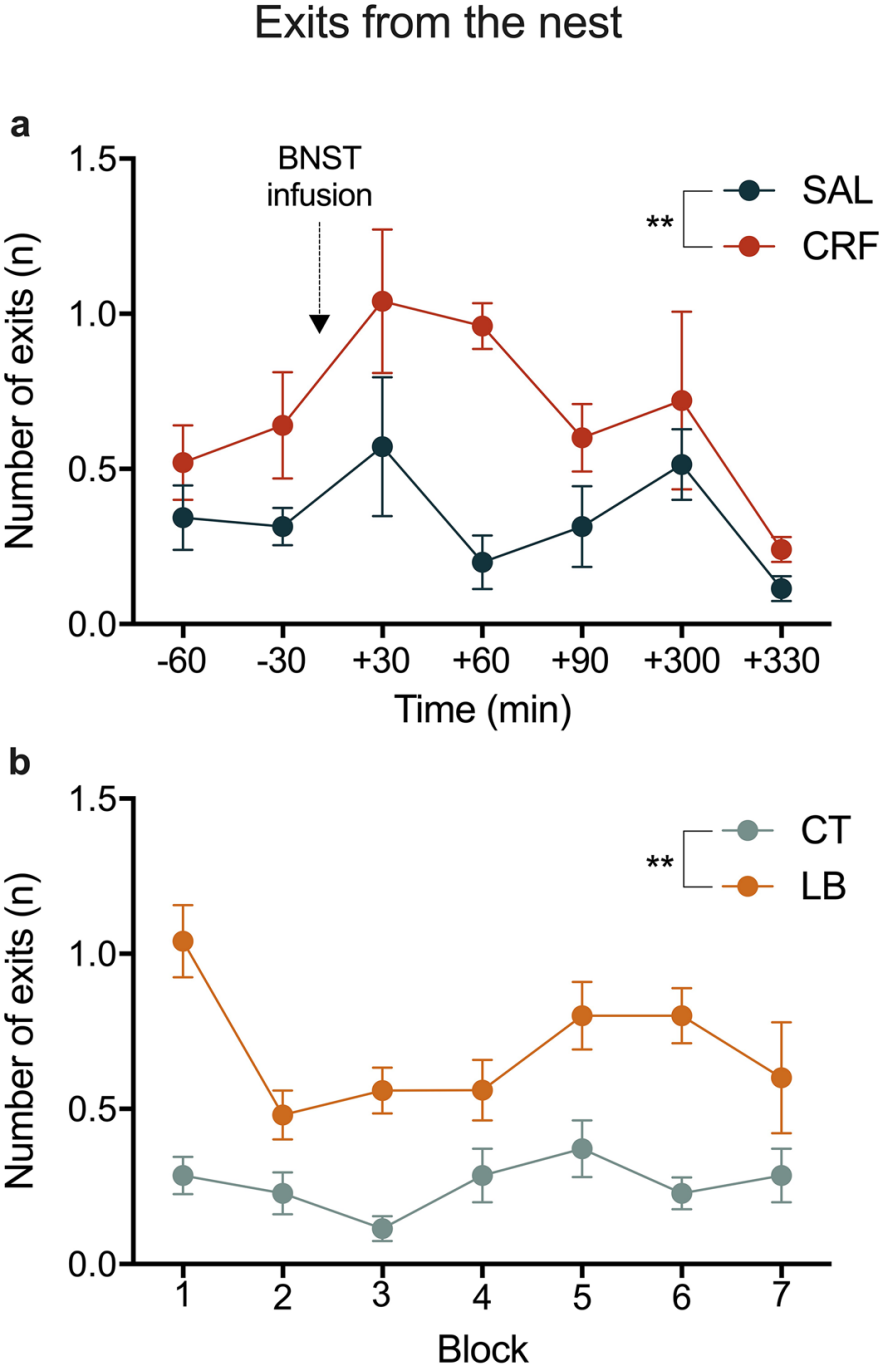
Maternal behaviour: Exits from the nest. (**a**) Frequency of exits from the nest in SAL and CRF dams per block. (**b**) Frequency of exits from the nest in CT and LB dams per block. Data are presented as means SEM of all days from each block. **p < 0.01 repeated-measures ANOVA, treatment effect. *n* = 5–7 dams per group. SAL, saline-infused dams; CRF, corticotropin-releasing factor-infused dams; CT, control dams; LB, stressed dams.

Regarding ABN behaviour, repeated-measures ANOVA showed a significant treatment effect, in which CRF treated dams showed a reduction in ABN compared to saline solution treated dams (SAL) [F (1, 10) = 21.385, *p* = 0.001; Fig. 3a]. Moreover, LB dams also presented a decrease in ABN compared to undisturbed control dams (CT) [F (1, 10) = 16.281, p = 0.002; Fig. 3b]. There was a significant treatment effect in relation to the number of exits from the nest, where CRF dams had an increased number of exits from the nest [F (1, 10) = 20.514, *p* = 0.001; Fig. 4a]. This pattern was also seen in LB dams compared to the pattern seen in CT dams [F (1, 10) = 218.788, *p* = 0.001; Fig. 4b]. Moreover, when comparing CT (non-infused) and SAL-treated groups, the two-way repeated-measures ANOVA showed no significant treatment or interaction effects [F (1,12) = 0.568, *p* = 0.465; F (1,12) = 0.91, *p* = 0.768, respectively]. These results indicate the absence of a saline infusion effect. No significant differences were detected in the other behaviours (Supplementary Figs. S1, S2 and S3).

### Offspring body weight

**Figure 5:**
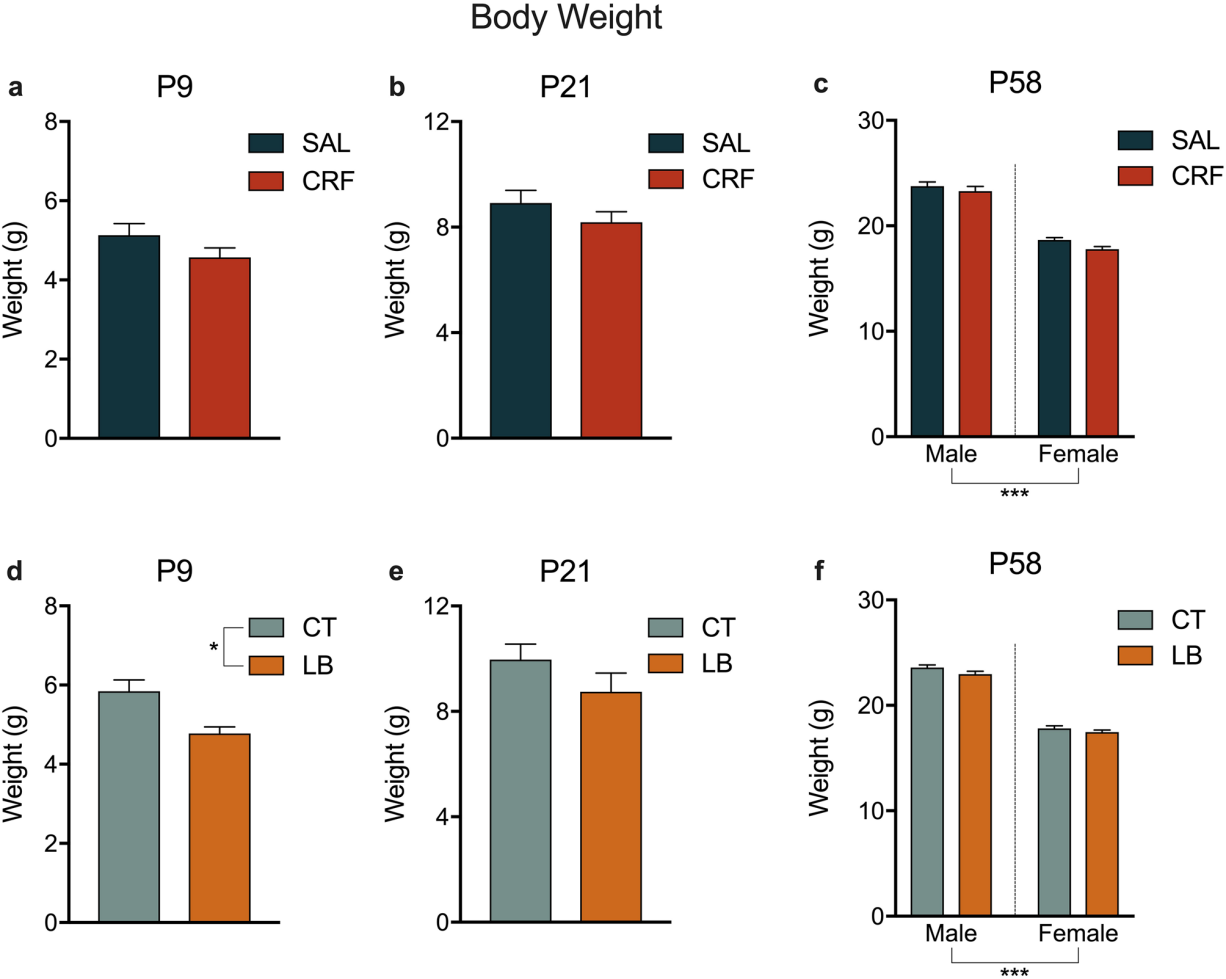
Body weight. (**a**) Body weight (in grams) of SAL and CRF offspring on P9. (**b**) Body weight (in grams) of SAL and CRF offspring on P21. (**c**) Body weight (in grams) of SAL and CRF offspring on P58. (**d**) Body weight (in grams) of CT and LB offspring on P9. (**e**) Body weight (in grams) of CT and LB offspring on P21. (**f**) Body weight (in grams) of CT and LB offspring on P58. Results are presented as means SEM. *p < 0.05, *t*-test; ***p < 0.001, two-way ANOVA, sex effect. *n* = 5–7 families per group (P9 and P21); *n* = 10–12 animals per group (P58). SAL, offspring of saline-infused dams; CRF, offspring of corticotropin-releasing factor-infused dams; CT, offspring of control dams; LB, offspring of stressed dams.

There were no significant differences in body weight between SAL and CRF mice on P9 or P21 (Fig. 5a,b, respectively). However, LB animals had reduced body weight at P9 compared to CT animals [t (14) = 2.391, p = 0.0314; Fig. 5d], but this difference was not detected on P21 (Fig. 5e). We identified a sex effect [F (1, 42) = 218.74, *p* < 0.0001; Fig. 5c; SAL × CRF] [F (1, 45) = 500.01, *p* < 0.0001; Fig. 5f; CT × LB] on P58; as expected, independent of the treatment, females presented a lower body weight than males.

### Anxiety-like behaviour tests

**Figure 6:**
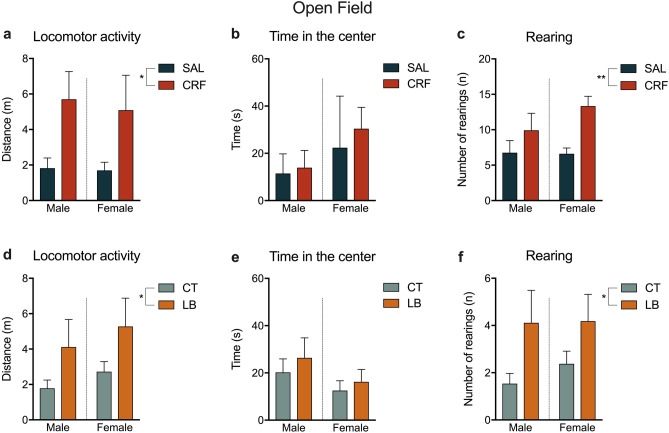
Parameters of the open field test. (**a**) Locomotor activity (in meters) of SAL and CRF offspring. (**b**) Time spent (in seconds) in the centre zone by SAL and CRF offspring. (**c**) Number of rearing behaviours by SAL and CRF offspring. (**d**) Locomotor activity (in meters) of CT and LB offspring. (**e**) Time spent (in seconds) in the centre zone by CT and LB offspring. (**f**) Number of rearing behaviours by CT and LB offspring. Results are presented as means SEM. * *p* < 0.05, two-way ANOVA, treatment-effect. *n* = 10–12 animals per group. SAL, offspring of saline-infused dams; CRF, offspring of corticotropin-releasing factor-infused dams; CT, offspring of control dams; LB, offspring of stressed dams.

We observed a significant treatment effect in the OF test, in which the offspring of CRF-infused dams showed an increase in the distance travelled [F (1, 38) = 7.216, *p* = 0.0107; Fig. 6a] compared to the offspring of SAL-infused dams. LB animals also presented increased locomotor activity compared to CT animals [F (1, 40) = 4.79, *p* = 0.034; Fig. 6d]. Moreover, CRF and LB animals increased the number of rearing behaviours in the OF test compared to SAL and CT animals, respectively [F (1,42) = 7.978, *p* = 0.0072; F (1, 45) = 6.696, *p* = 0.0130; Fig. 6c,f]. In both SAL × CRF and CT × LB groups, no significant difference was detected in the time spent in the centre zone of the OF (Fig. 6b,e) and in the number of stretching and self-grooming behaviours (Supplementary Table S1).

**Figure 7:**
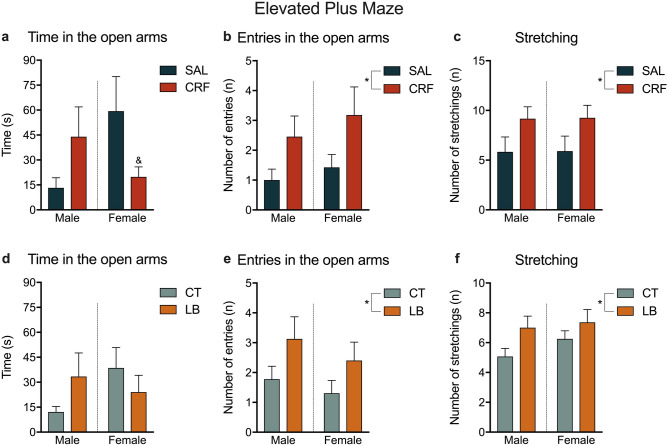
Parameters of the elevated plus-maze. (**a**) Time spent (in seconds) in the open arms by SAL and CRF offspring. (**b**) Number of entries into the open arms by SAL and CRF offspring. (**c**) Number of stretching behaviour by SAL and CRF offspring. (**d**) Time spent (in seconds) in the open arms by CT and LB offspring. (**e**) Number of entries into the open arms by CT and LB offspring. (**f**) Number of stretching behaviour by CT and LB offspring. Results are presented as means SEM. Two-way ANOVA, interaction-effect, where CRF males increased their time while CRF females decreased. *p < 0.05, 2-way ANOVA, treatment-effect. *n* = 10–12 animals per group. SAL, offspring of saline-infused dams; CRF, offspring of corticotropin-releasing factor-infused dams; CT, offspring of control dams; LB, offspring of stressed dams.

In the EPM test, a significant interaction effect was found in the time spent in the open arms, where males from CRF-infused dams spent increased time, whereas females spent less time exploring the open arms [F (1, 41) = 6.267, *p* = 0.0164; Fig. 7a]. Moreover, there was a significant treatment effect in the number of entries into the open arms and stretching behaviour, where male and female offspring of CRF-infused dams had more entries into the open arms and performed more stretching behaviour than those of SAL-infused dams [F (1, 37) = 5.3, *p* = 0.0271; F (1, 42) = 5.8, *p* = 0.0196; Fig. 7b,c, respectively]. LB males and females also showed an increase in the number of entries into the open arms and stretching behaviour compared to CT animals [F (1, 36) = 4.821, *p* = 0.0346; F (1, 46) = 5.1, *p* = 0.0287; Fig. 7e,f, respectively]. No significant differences were observed in the number of head dipping behaviour and in self-grooming behaviour in CRF and LB animals compared to SAL and CT animals, respectively (Supplementary Table S1). Moreover, no significant differences were observed between LB and CT animals on the time spent in the open arms (Fig. 7d).

**Figure 8:**
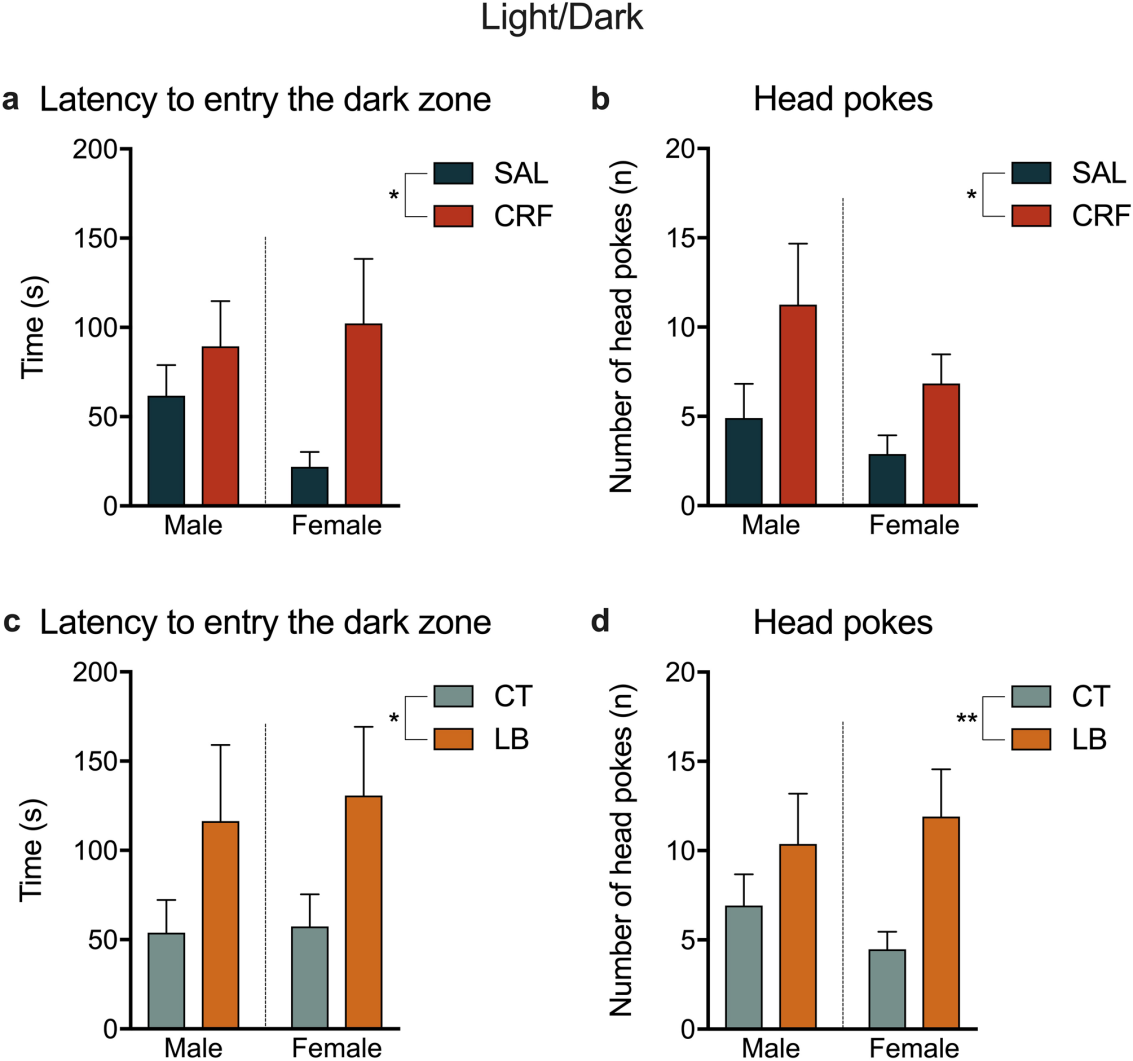
Parameters of the light/dark test. (**a**) Latency (in seconds) to enter the dark zone of SAL and CRF offspring. (**b**) Number of head pokes by SAL and CRF offspring. (**c**) Latency (in seconds) to enter the dark zone of CT and LB offspring. (**d**) Number of head pokes by CT and LB offspring. Results are presented as means SEM. *p < 0.05, **p < 0.01, two-way ANOVA, treatment-effect*. n* = 10–12 animals per group. SAL, offspring of saline-infused dams; CRF, offspring of corticotropin-releasing factor-infused dams; CT, offspring of control dams; LB, offspring of stressed dams.

There was a significant treatment effect in the LD test, in which animals of the CRF group took longer time to enter the dark zone [F (1, 40) = 4.316, *p* = 0.0442; Fig. 8a] and showed an increased number of head pokes from the light to the dark zone than those of the SAL group [F (1, 40) = 4.807, *p* = 0.0342; Fig. 8b]. Stressed animals from the LB group showed similar results, in which they took longer to enter the dark zone [F (1, 22) = 4.881, *p* = 0.0379 Fig. 8c] and showed increased number of head pokes into the dark zone [F (1, 42) = 7.455, *p* = 0.0092; Fig. 8d] than CT animals. No significant differences were observed in the remaining measures of the LD (Supplementary Table S1).

## Discussion

We demonstrated that CRF infusion in the BNST of dams during the first weeks after birth altered maternal care by decreasing ABN and increasing the number of exits from the nest. These changes in maternal care resulted in increased anxiety-like behaviour among female offspring, while it was decreased among male offspring. Higher locomotor activity was observed in both male and female offspring of CRF- and LB-exposed dams. Although this was a sex-specific effect, the present study suggests that altered maternal care is an important factor when accounting for the effects of rodent models of early life stress. Additionally, we have shown that these behavioural alterations resemble those observed in offspring raised in a stressful environment elicited by LB exposure.

Anxiety-like behaviour can be assessed using different protocols. In this study, we opted to use the classic ones: the OF, EPM, and LD tests^35^. Standardized measures such as the time spent in the open arms of the EPM or in the centre of the OF are most often reported when investigating anxiety-like behaviour. However, ethological measures are also important to fully comprehend how an intervention can impact a rodent behaviour^36^. With that in mind, we also analysed risk assessment, rearing, stretching and self-grooming behaviour as ethological measures. In the OF test, CRF and LB animals increased their total distance travelled compared to SAL and CT animals, respectively. This is a well-known behavioural measure of locomotor activity and exploration, and our results are congruent with those found by Aya-Ramos et al.^37^ and Wang et al.^38^, who found that measures of locomotor activity increase significantly in animals subjected to postnatal maternal separation. Correspondingly, CRF and LB animals increased the number of rearing behaviours compared to its respective controls, which is associated with locomotion and exploration^39^. Nevertheless, we observed no change in the time spent in the centre zone, which is a classical measure of anxiety-like behaviour in this test.

In the EPM test, CRF and LB animals increased the number of stretching behaviours and entries into the open arms, which are related to exploratory actions, corroborating the OF test results. The most classic anxiety-related parameter in the EPM test is the time spent in the open arms, in which less time exploring open compartments is an indication of more anxiety-like behaviour^40^. Intriguingly, we observed a significant interaction effect, where CRF males spent more time in the open arms, whereas CRF females spent less time compared to SAL animals, but no difference in this regard was observed between CT and LB animals. This interaction is in agreement with previous studies that showed an anxiolytic effect of maternal separation in male offspring^41^. In addition, it also agrees with previous studies showing that female animals are more vulnerable to the effects of early life stress in a wide range of behavioural and neurobiological outcomes^42,43^. According to Keller et al.^44^, the increased vulnerability in females may be related to impaired maternal care. These authors showed that in a maltreatment condition, female offspring received higher adverse care from the dams than male offspring. Additionally, Bowers et al.^45^ presented evidence that male pups, when separated from their mothers, emitted more ultrasonic vocalisations than female pups, and the dams were more likely to retrieve the male pups first. With this data, we suppose that due to the increased vocalisation, males receive more maternal care than females and, therefore, could be better protected against early life stress effects, thereby presenting less anxiety-like behaviour phenotypes later in life. In the LD test, we showed that CRF and LB animals took longer to enter the dark zone and increased the number of head pokes into the transition from the light to the dark zone than SAL and CT animals. These behaviours suggest a stronger link to risk assessment and exploratory actions, corroborating the OF test results. However, we could not observe differences in the time spent in each chamber and the total number of transitions, which are considered classic measures of the LD test.

Behavioural alterations observed in the offspring could be elicited by activation of the CRF-BNST signalling pathway in the limbic system of dams. As mentioned, CRF expression activates the HPA axis, increasing the release of glucocorticoids^28^. For this reason, CRF infusion in the BNST has been associated with different behavioural outcomes such as anxiogenic-like effects and involvement in the fear response^46–49^. CRF in the BNST also plays a role in social-defeat and anhedonia^29,50^. Although there is growing evidence suggesting that the CRF signalling in the BNST has an important role in maternal care^51^ there are other regions strongly related to this behaviour, such as the hypothalamic medial preoptic area (mPOA). To be noted, the mPOA and its adjacent projections to the BNST form what is known as a “super-core” brain hub that regulates maternal care^52^. In this sense, a recent study from Klampfl et al.^53^ presented the effects of CRFR1 agonist infusion in the mPOA on maternal care and maternal anxiety-like behaviour. The study evidenced that the infusion of CRFR1 in the mPOA leads to a decrease in ABN, and also to an increase in off-nest behaviours and anxiety-like phenotypes. Infused dams also presented increased expression of oxytocin in the mPOA. The oxytocin is a neuropeptide highly expressed during lactation; its receptor is present in different regions including the BNST and the mPOA^54^. This increase in oxytocin expression may be a tentative mechanism triggered to counteract the effects of CRF overexpression. However, even though the oxytocin is extremely important to the onset of maternal care it does not enhance ongoing maternal care^55^. Although the assessment of oxytocin goes beyond the scope of this study, one possibility is that the BNST and the mPOA act together in the regulation of those behaviours through CRF and oxytocinergic signalling. It also should be noted that the BNST is highly interconnected with the central amygdala, which mediates key behavioural changes triggered by stress exposure^56^. As mentioned, CRF injection into the BNST may elevate general anxiety levels, thereby altering maternal behaviour and anxiety-like behaviour in the offspring. However, this might indicate an indirect involvement of this nucleus in mediating specific maternal behaviours. Therefore, our findings should be carefully interpreted, particularly considering that maternal behaviours have been reported to be more dependent on ventral area (below the anterior commissure) of the BNST sub-nuclei^57^.

Several studies that applied the LB protocol reported that stressed LB dams increased the number of exits from the nest compared to CT dams^18,20,58–61^ which correspond with our results, as CRF and LB dams increased their nest exits compared to SAL and CT dams, respectively. This augmentation in the frequency of exits from the nest alters the natural pattern of maternal behaviour, resulting in fragmented maternal care, due to the increased number of times that the dam interrupts nurturing behaviour. We also observed a decrease in the frequency of ABN in CRF dams compared to that in SAL dams. However, our ABN results do not totally correspond to those of other studies with early life stress protocols. Most studies that used maternal separation report an increase in this behaviour after reunion in the mothers^62–65^, probably as an attempt to compensate for the time away from the pups. Unfortunately, there is a lack of information about maternal behaviour before and after a longer period before reunion. Although these maternal separation studies reported an increased ABN, they also reported anxiety-like behaviour in the offspring of these stressed dams, so we suppose that the compensatory nursing behaviour post-reunion is not effective in protecting pups from further consequences.

Certain limitations should be considered when interpreting the present study’s findings. First, we did not measure specific anxiety and depression-like behavior in the dams during the infusion period. Moreover, the same offspring cohort of animals was used for the behaviour battery. However, in order to address the cumulative stress issue, we performed the tests from less to more stressful, and all groups went through the same protocol. Next, this study tested only one dose of CRF. This dose was chosen due to our intent to replicate previously presented results^23^. Finally, the technique used for the infusions did not allow us to measure the degree of CRF spread to the surrounding regions of the BNST. Even though we cannot exclude this possibility, it is unlikely that the CRF spread to subjacent areas since we used a slow injection rate (0.1 ul/min) for a small amount of drug (1 μg/0.5 μL).

Therefore, we provided additional evidence to the hypothesis that poor maternal care during early life anticipates altered behavioural phenotypes in rodents during adolescence and adulthood, including changes in exploratory and anxiety-like behaviours. Although the exact mechanisms underlying these outcomes and the sex-specific effect remain unknown, it is important to note that sex is a key factor to alterations in exploratory and anxiety-like behaviours elicited by poor mother-infant attachment.

## Material and methods

### Animals

This study was conducted with BALB/cJ mice from the colony of the Center for Experimental Biological Models (CeMBE) at Pontifical Catholic University of Rio Grande do Sul, Brazil. Animals were maintained under a 12-h/12-h light–dark cycle in ventilated Plexiglas cages with room temperature controlled at 21 1C and access to food and water ad libitum. The cages were changed once per week, except during P0-9. Mice were bred in-house. Female BALB/cJ mice were pair-housed with male BALB/cJ mice for 48 h, and then males were moved to a new cage. After 17 days, females were checked daily for the presence of pups. The day of birth was considered P0. We used 24 litters in total: saline (SAL; n = 7), CRF (n = 5), control (CT; n = 7), LB (n = 5). Pups were weighed on P9, P21, and P58 and weaned on P21. After weaning, 3–5 animals of the same sex were grouped per cage and left undisturbed, except for cage cleaning, until the test day (P58). Mice were sacrificed 30 min after the last behavioural test on P60. The procedures included in this study were conducted in accordance with the Guide for the Care and Use of Laboratory Animals from the National Institute of Health (NIH) and were approved by the Ethics Committee on the Use of Animals of Pontifical Catholic University of Rio Grande do Sul, under the protocol code 9080. Figure 1 displays the experimental design of the study (created with BioRender.com).

### Stereotaxic surgery and infusions

Adult females (~ P60) from the SAL and CRF groups were implanted with a dual-cannula system (P1 Technologies; Roanoke, VA) targeting the BNST under intraperitoneal anaesthesia using a combination of ketamine (100 mg/kg) and xylazine (10 mg/kg). As per the mouse brain stereotaxic coordinate atlas^66^, the coordinates were + 0.3 mm posterior to the bregma, 1.1 mm lateral to the midline, and 4.3 mm ventral to the dura. CRF (1 μg/0.5 μL, CRF human/rat, Tocris Bioscience; Bristol, United Kingdom) and SAL (0.5 μL, 0.9% NaCl) were infused at 0.1 μL/min using an infusion pump (Accu-Chek Insight, Roche; Basel, Switzerland). Doses were chosen based on previous studies^23,34,67^. To perform the infusions, dams were removed from their home cage and transported into a clean cage to the infusion room, where they were quickly immobilised. Injectors were inserted into the cannula, and the dam was able to freely move during the infusion. Infusions were repeated five times on P1, P3, P5, P7, and P9. To verify the correct placement of the cannula, the dams were perfused on the day of weaning. The brains were cut into 30-μm coronal sections, mounted on slides, and stained with haematoxylin and eosin (Fig. 2a,b).

### LB protocol

The LB protocol was adapted from Rice et al.^18^. From P2 to P9, CT dams and offspring were placed in cages with standard amounts of wood shavings, while LB dams and offspring were placed in cages with 1 g of cotton and an aluminium mesh above the wood shavings. After P9, the LB dams and litters were placed in new cages prepared according to CT conditions.

### Maternal behaviour observations

The maternal behaviour observation protocol was adapted from Klampfl et al.^23^. The protocol was conducted on P1, P3, P5, P7, and P9 (on days of SAL/CRF infusions). Observations were conducted in seven blocks of 30 min each: before the infusion (~ 08:00–09:00, blocks 1 and 2), 30 min after infusion (~ 09:30–11:00; blocks 3, 4, and 5), and the last 5 h after infusion (~ 14:00–15:00, blocks 6 and 7). The same days and hours were used to observe the maternal behaviour of CT and LB dams. One observation was made every 2 min for 105 observations per day. The frequency of the following behavioural categories was evaluated: ABN, blanket or passive nursing (N), licking and grooming (L), off nest (X), self-grooming (S), eating or drinking (E), and the number of exits from the nest (EN). Maternal behaviour was recorded by two independent observers. Results are presented as the mean of all days from each block.

### Anxiety-like behaviour tests

OF, EPM, and LD tests were performed on P58, P59, and P60, respectively. All tests were video-recorded and analysed using the ANY-maze software (Stoelting; Wood Dale, IL). All tests were performed during the light phase. The apparatus was cleaned with 70% ethanol before testing the next animal.

### OF test

The OF apparatus consisted of a clear Plexiglas box (33 cm × 33 cm), and the test was performed under 140–150 lx illumination. Mice were placed in the middle of the apparatus and allowed to explore for 10 min. The box was divided into 16 squares: the central four squares composed the centre zone, and the remaining squares composed the periphery zone. We evaluated the time spent in the centre and periphery zones, the total distance travelled and the number of instances of rearing, stretching and self-grooming behaviour.

### EPM test

The EPM apparatus consisted of black Plexiglas, with two open arms (30 cm × 5 cm each) and two closed arms (30 cm × 5 cm × 15 cm) connected by a central area (5 cm × 5 cm), at a height of 50 cm above the ground, illuminated at 40 lx. The animal was placed in the central area facing an open arm and was allowed to explore for 5 min. Total time, the number of entries in each arm, and the number of instances of head dipping (risk assessment measure, when the animal lean its head out of the open arm) and self-grooming were analysed.

### LD test

The apparatus used for the LD test was constructed with Plexiglas (21 cm × 42 cm × 20 cm), divided in two identical-sized chambers connected by a door. One side was illuminated at 390 lx, and the other was completely dark. The animal was placed in the bright chamber and allowed to explore for 10 min. The total time spent in each chamber, latency to first entry into the dark chamber, number of transitions, head pokes (risk assessment measure, when the animal lean its head through the connection door) the animal did to enter or exit the dark zone and the number of instances of rearing and self-grooming behaviour were analysed.

### Statistical analysis

All statistical analyses were performed using SPSS version 23.0 (IBM; Armonk, NY), and graphs were constructed using GraphPad Prism 8 (GraphPad Software; San Diego, CA). Data are presented as means standard error of the mean (SEM). Statistical significance was considered when *p* < 0.05. Body weight (P9 and P21) was analysed using Student’s t-test. Repeated-measures analysis of variance (ANOVA) and multivariate analysis of variance (MANOVA) were used to analyse maternal behaviour. Two-way ANOVA was used for behavioural tests (OF, EPM, LD) and body weight (P58), with a sex factor (male × female) and a treatment or condition factor (SAL × CRF or CT × LB, respectively). ANOVAs were followed by Tukey’s post-hoc tests.

## Supplementary information

Supplementary Information 1.
