# Supplementary material for: Corticotropin-releasing factor infusion in the bed nucleus of the stria terminalis of lactating mice alters maternal care and induces behavioural phenotypes in offspring
Source: Sci Rep. 2020 Nov 17;10:19985. doi: 10.1038/s41598-020-77118-7 (PMC7672063; doi:10.1038/s41598-020-77118-7)
Supplement: Supplementary file 1 — Supplementary Information 1. [file 41598_2020_77118_MOESM1_ESM.docx]

**Corticotropin-releasing factor infusion in the bed nucleus of the stria terminalis of lactating mice alters maternal care and induces behavioural phenotypes in offspring**

Kerstin Camile Creutzberg^1^, Érika Kestering-Ferreira^1^, Thiago Wendt Viola^1^, Luis Eduardo Wearick-Silva^1^, Rodrigo Orso^1^, Bernardo Aguzzoli Heberle^1^, Lucas Albrechet-Souza^2^, Rosa Maria Martins de Almeida^3^, Rodrigo Grassi-Oliveira^1^.

**Supplementary Material**

**
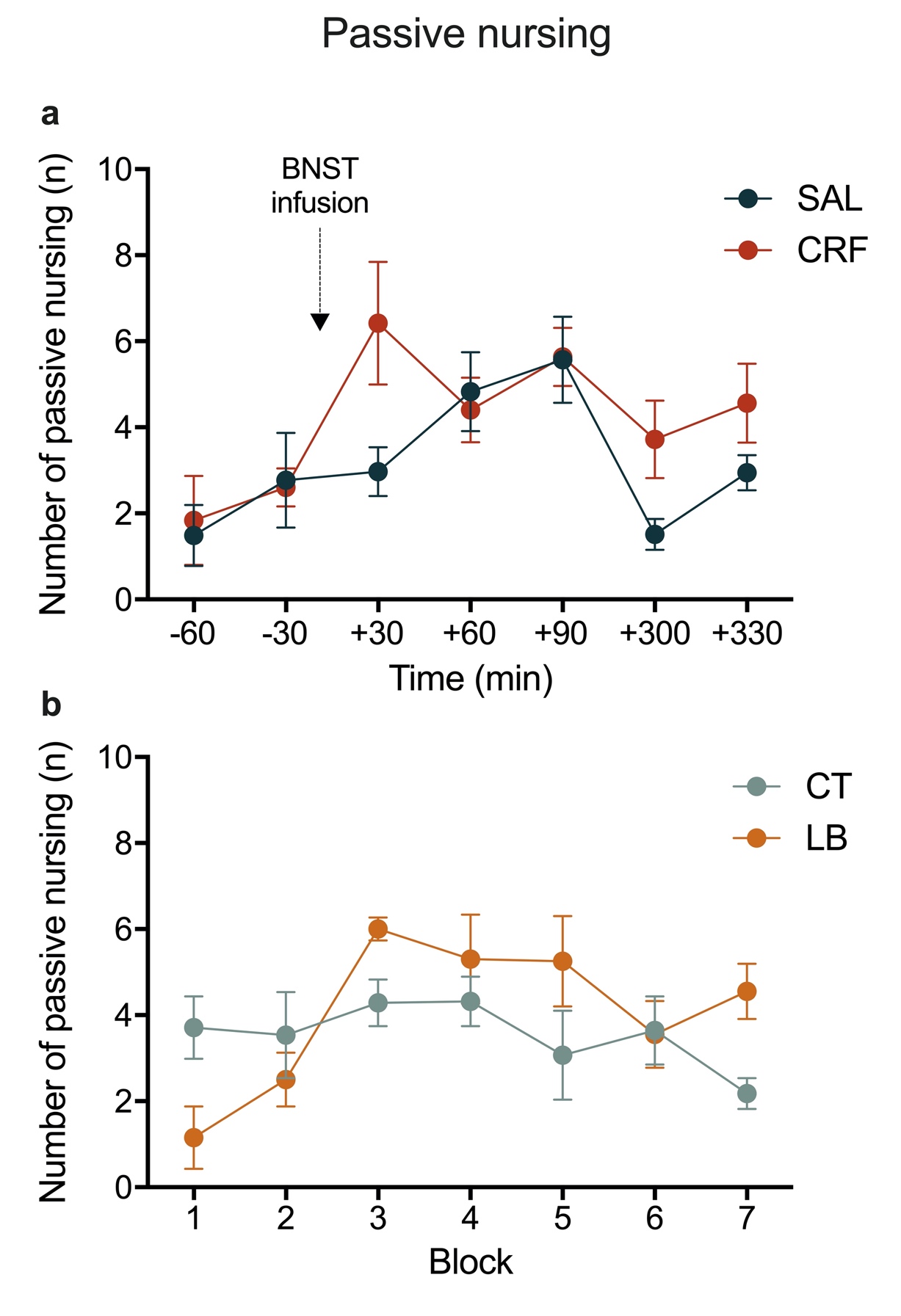
**

**Fig. S1.** Maternal behaviour: Passive nursing. (a) Frequency of passive nursing in SAL and CRF dams per block. (b) Frequency of passive nursing in CT and LB dams per block. Data are presented as means ± SEM of all days from each block. *n* = 5-7 dams per group. SAL, saline-infused dams; CRF, corticotropin-releasing factor-infused dams; CT, control dams; LB, stressed dams; ABN, arched-back nursing.

**
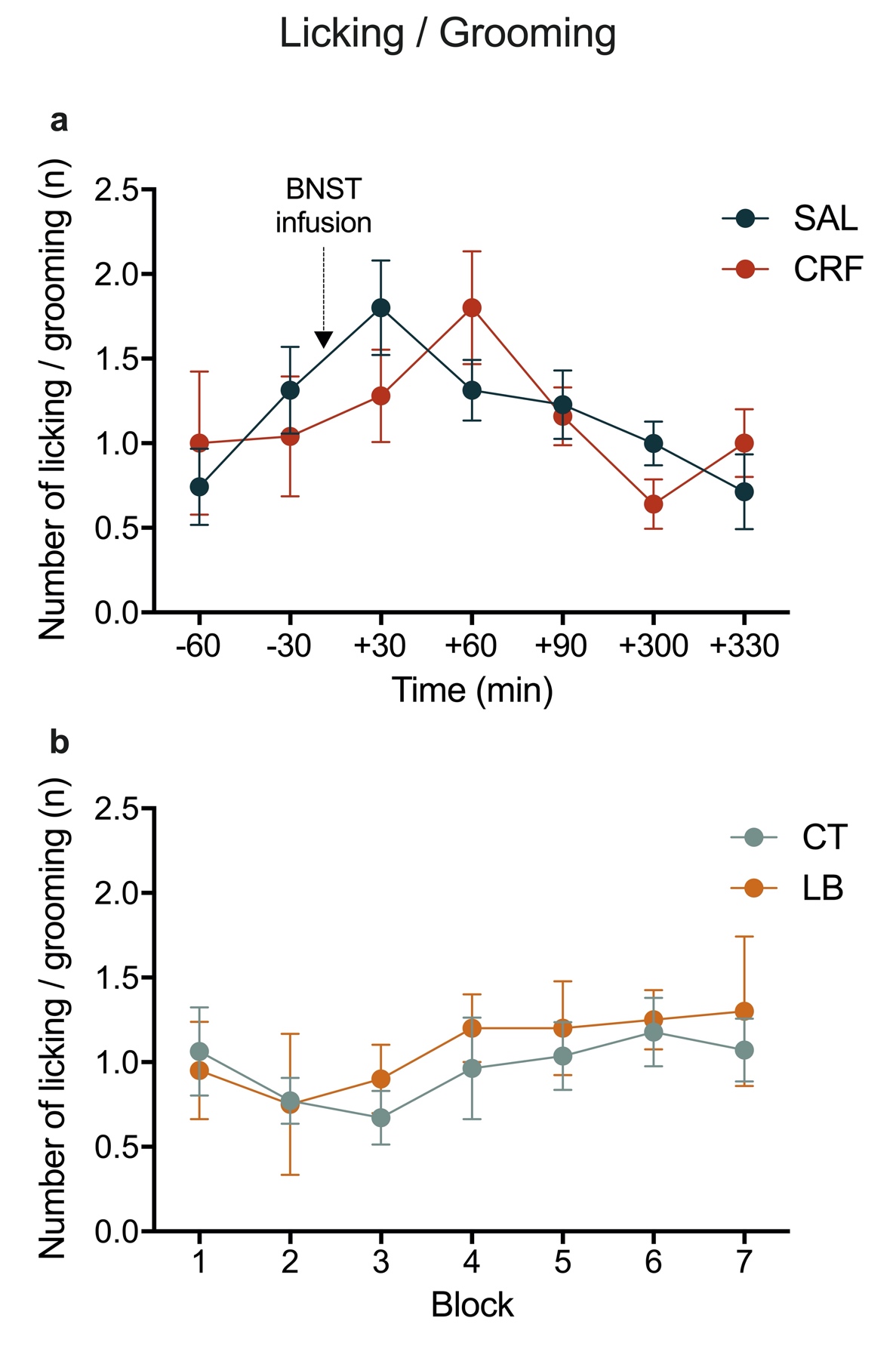
**

**Fig. S2.** Maternal behaviour: Licking / Grooming. (a) Frequency of licking / grooming in SAL and CRF dams per block. (b) Frequency of licking / grooming in CT and LB dams per block. Data are presented as means ± SEM of all days from each block. *n* = 5-7 dams per group. SAL, saline-infused dams; CRF, corticotropin-releasing factor-infused dams; CT, control dams; LB, stressed dams.


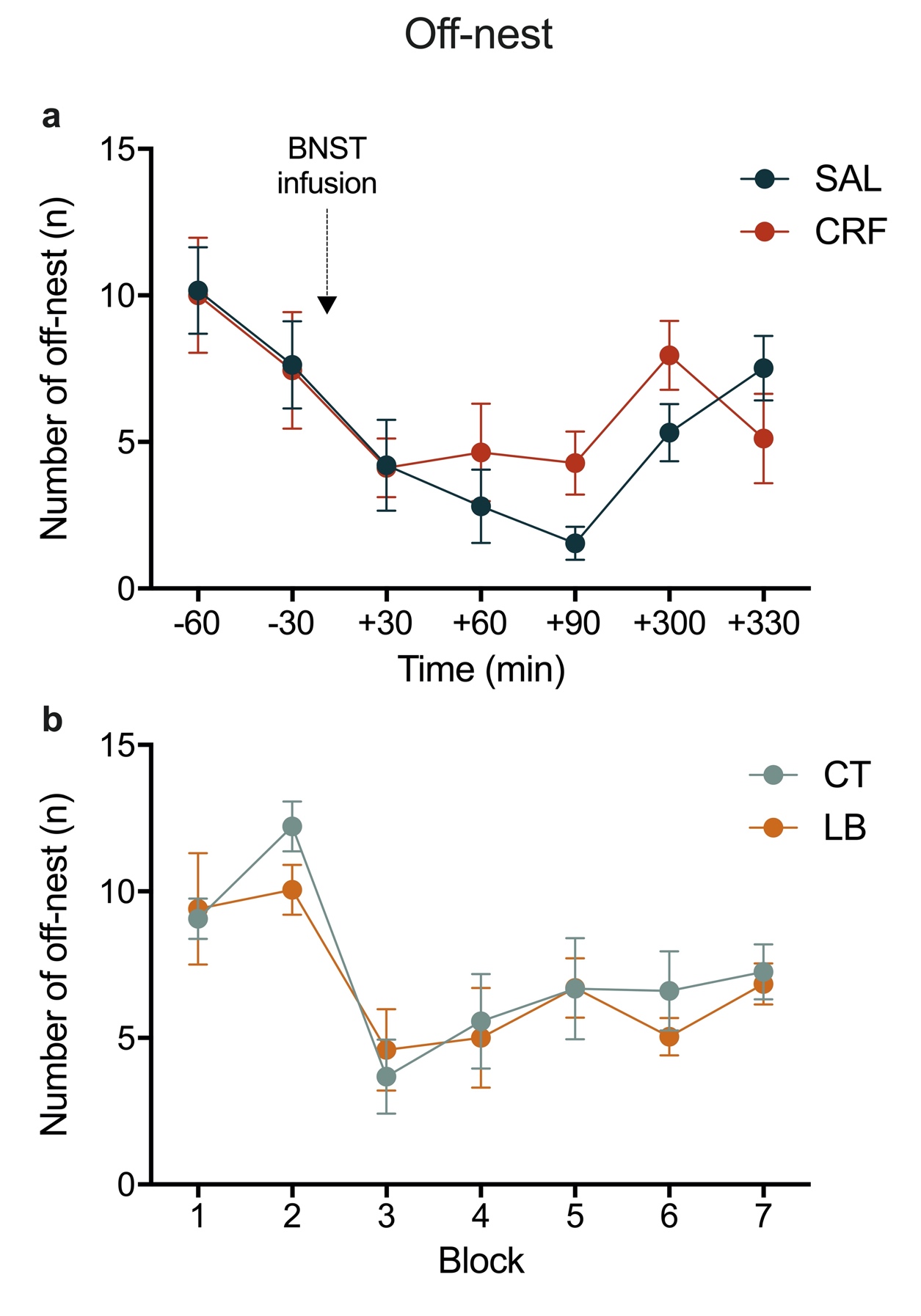


**Fig. S3.** Maternal behaviour: Off-nest. (a) Frequency of off-nest in SAL and CRF dams per block. (b) Frequency of off-nest in CT and LB dams per block. Data are presented as means ± SEM of all days from each block. *n* = 5-7 dams per group. SAL, saline-infused dams; CRF, corticotropin-releasing factor-infused dams; CT, control dams; LB, stressed dams.

**Table S1**. Anxiety-like behaviour measures.

| **Open Field Test** | **Mean ± SD SAL male** | **Mean ± SD SAL female** | **Mean ± SD CRF male** | **Mean ± SD CRF female** | ***p***  **Interaction** | ***p***  **Sex effect** | ***p***  **Treatment effect** |
| --- | --- | --- | --- | --- | --- | --- | --- |
| Stretching (n) | 26.91 ± 10.86 | 28.6 ± 12.53 | 24.5 ± 14.95 | 31 ± 17.8 | 0.573 | 0.340 | 0.998 |
| Self-grooming (n) | 6.16 ± 4.42 | 7.4 ± 5.71 | 10.91 ± 5.82 | 8.41 ± 5.79 | 0.254 | 0.696 | 0.081 |
|  | **Mean ± SD CT male** | **Mean ± SD CT female** | **Mean ± SD**  **LB male** | **Mean ± SD**  **LB female** |  |  |  |
| Stretching (n) | 28.71 ± 14.05 | 29.37 ± 9.83 | 23.22 ± 5.89 | 34.63 ± 12.5 | 0.107 | 0.071 | 0.972 |
| Self-grooming (n) | 9.78 ± 3.16 | 9.81 ± 4.9 | 9 ± 2.54 | 12.81 ± 5.11 | 0.124 | 0.119 | 0.364 |

| **Elevated Plus Maze Test** | **Mean ± SD SAL male** | **Mean ± SD SAL female** | **Mean ± SD CRF male** | **Mean ± SD CRF female** | ***p***  **Interaction** | ***p***  **Sex effect** | ***p***  **Treatment effect** |
| --- | --- | --- | --- | --- | --- | --- | --- |
| Self-grooming (n) | 2.66 ± 2.64 | 2 ± 2.74 | 3.75 ± 3.13 | 3.5 ± 2.64 | 0.802 | 0.583 | 0.126 |
| Head dips (n) | 27.72 ± 15.12 | 24.5 ± 12.92 | 25.18 ± 14.44 | 23.91 ± 16.39 | 0.828 | 0.619 | 0.729 |
|  | **Mean ± SD CT male** | **Mean ± SD CT female** | **Mean ± SD**  **LB male** | **Mean ± SD**  **LB female** |  |  |  |
| Self-grooming (n) | 3.64 ± 3.34 | 2.93 ± 2.14 | 3.88 ± 2.08 | 4.27 ± 2.9 | 0.488 | 0.837 | 0.316 |
| Head dips (n) | 18 ± 10.2 | 13.43 ± 5.68 | 17.66 ± 4.92 | 14.72 ± 6.48 | 0.703 | 0.082 | 0.822 |

| **Light/Dark Test** | **Mean ± SD SAL male** | **Mean ± SD SAL female** | **Mean ± SD CRF male** | **Mean ± SD CRF female** | ***p***  **Interaction** | ***p***  **Sex effect** | ***p***  **Treatment effect** |
| --- | --- | --- | --- | --- | --- | --- | --- |
| Time spent in the light (sec) | 87.94 ± 62.17 | 82.88 ± 39.30 | 103.07 ± 67.68 | 81.76 ± 63.74 | 0.712 | 0.550 | 0.750 |
| Transitions (n) | 1.37 ± 1.4 | 1.09 ± 1.13 | 2.6 ± 2.17 | 1.77 ± 1.85 | 0.628 | 0.322 | 0.091 |
| Rearing (n) | 0.75 ± 1.42 | 0.8 ± 2.2 | 0.5 ± 1.16 | 0.75 ± 1.05 | 0.821 | 0.735 | 0.735 |
| Self-grooming (n) | 4.41 ± 6.58 | 5.3 ± 5.73 | 2.41 ± 4.37 | 2.5 ± 2.61 | 0.789 | 0.746 | 0.113 |
|  | **Mean ± SD CT male** | **Mean ± SD CT female** | **Mean ± SD**  **LB male** | **Mean ± SD**  **LB female** |  |  |  |
| Time spent in the light (sec) | 120.1 ± 24.12 | 95.37 ± 45.18 | 119.38 ± 51.42 | 116.68 ± 51.79 | 0.608 | 0.524 | 0.632 |
| Transitions (n) | 3 ± 2.92 | 2.53 ± 2.64 | 2.75 ± 1.98 | 2.90 ± 2.62 | 0.694 | 0.847 | 0.937 |
| Rearing (n) | 0.85 ± 1.35 | 0.06 ± 0.25 | 0.44 ± 0.72 | 0.63 ± 1.02 | 0.072 | 0.267 | 0.765 |
| Self-grooming (n) | 5 ± 4.42 | 2.62 ± 3.44 | 3.11 ± 3.68 | 4.63 ± 3.07 | 0.076 | 0.695 | 0.954 |

Note: *p* = significance level two-way ANOVA.
